# Norspermidine and Novel Pd(II) and Pt(II) Polynuclear Complexes of Norspermidine as Potential Antineoplastic Agents Against Breast Cancer

**DOI:** 10.1371/journal.pone.0055651

**Published:** 2013-02-13

**Authors:** Tânia Magalhães Silva, Sara Andersson, Sunil Kumar Sukumaran, Maria Paula Marques, Lo Persson, Stina Oredsson

**Affiliations:** 1 Research Unit “Molecular Physical-Chemistry”, University of Coimbra, Portugal; 2 Department of Biology, University of Lund, Sweden; 3 Department of Experimental Medical Science, University of Lund, Sweden; 4 Anthem Biosciences Pvt. Ltd., 49 Canara Bank Road, Bangalore, India; 5 Departament of Life Sciences, Faculty of Science and Technology, University of Coimbra, Portugal; AMS Biotechnology, United Kingdom

## Abstract

**Background:**

New strategies are needed for breast cancer treatment and one initial step is to test new chemotherapeutic drugs in breast cancer cell lines, to choose candidates for further studies towards clinical use.

**Methodology and Findings:**

The cytotoxic effects of a biogenic polyamine analogue – norspermidine – and its trinuclear Pd(II) and Pt(II) complexes – Pd_3_NSpd_2_ and Pt_3_NSpd_2_, respectively – were investigated in one immortalized normal-like and three breast cancer cell lines. The normal-like MCF-10A cells were least sensitive to the compounds, while growth inhibition and cell death was observed in the cancer cell lines. Norspermidine and its Pd(II) complex were generally shown to have stronger antiproliferative effects than the corresponding Pt(II) complex. Moreover, both norspermidine and the Pd(II) complex reduced the cellular activity of the growth-related enzyme, ornithine decarboxylase (ODC) to a lower level than the Pt(II) complex in most of the cell lines examined. Treatment with norspermidine or the Pd(II) complex reduced the number of colonies formed in a soft agar assay performed with the breast cancer cell lines, indicating that these compounds reduced the malignancy of the breast cancer cells. The effect of norspermidine or the Pd(II) complex on colony formation was much stronger than that observed for the Pt(II) complex. The results from a new mammalian genotoxicity screen together with those of a single cell gel electrophoresis assay indicated that none of the drugs were genotoxic at a 25 µM concentration.

**Main Conclusions:**

Overall, norspermidine and its Pd(II) complex were shown to have strong antiproliferative effects. In comparison, the effects obtained with the Pd(II) complex were much stronger than that of the Pt(II) complex. The results obtained in the present study demonstrate that the trinuclear Pd(II) complex of norspermidine (Pd_3_NSpd_2_) may be regarded as a potential new metal-based drug against breast cancer, coupling a significant efficiency to a low toxicity.

## Introduction

Despite advances in the detection and treatment of breast cancer, this is still one of the most widely spread malignant tumor forms among women [1]. Therefore, it is essential to search for and test the efficiency of new potential drugs, in order to obtain new chemotherapeutic candidates for development towards clinical use [2], [3]. Testing in breast cancer cell lines is one early means of investigating new compounds for anticancer activity.

The naturally occurring polyamines spermidine (H_2_N(CH_2_)_3_NH(CH_2_)_4_NH_2_), spermine (H_2_N(CH_2_)_3_NH(CH_2_)_4_NH(CH_2_)_3_NH_2_) and their diamine precursor putrescine (H_2_N(CH_2_)_4_NH_2_) are positively charged substances at physiological pH and virtually present in all prokaryotic and eukaryotic cells [4]–[6]. Because of their cationic nature, the polyamines can interact with negatively charged molecules within the cell, such as DNA, RNA, proteins and phospholipids, thereby affecting their structure and function [7].

Biogenic polyamines are crucial for a variety of cellular processes including proliferation, differentiation and apoptosis [7]. The intracellular polyamine pool is limited, at the lower level, by its requirement for cell survival and proliferation and, at the upper limit, by its cytotoxicity, which may induce cell death [8]. Due to their importance in essential cellular functions, there is a strict control of the intracellular levels of polyamines via multiple regulatory mechanisms affecting biosynthesis, catabolism, uptake and excretion [8], [9].

Since polyamines have been shown to affect numerous processes in carcinogenesis, their metabolic pathways are highly interesting as potential targets for novel antitumor drugs [10]. Thus, specific inhibitors of the enzymes involved in either the polyamine biosynthetic or catabolic pathways, as well as synthetic polyamine analogues have been developed as potential anticancer agents [11]–[15].

Some of the polyamine analogues are efficiently taken up by cells through the natural polyamine transport system. Once inside the cell, where they can accumulate to high concentrations, they cannot functionally substitute for the natural polyamines due to structural differences (*e.g.* alkylation at the terminal nitrogens, which is known to be an essential site for activity). Therefore, many of these polyamine analogues stimulate catabolism and down-regulate biosynthesis of the biogenic polyamines, leading to the depletion of the intracellular polyamine pool. This will cause the disruption of several cellular processes and may constitute a promising anticancer strategy [11], [16], [17]. In fact, there are several polyamine analogues presently being tested in clinical trials as new chemotherapeutic agents [14], [18], [19].

Platinum(II) and palladium(II) antitumor compounds are known to be cytotoxic through covalent binding to DNA (mainly to the N_7_ atom of the purine bases), yielding both intra- and interstrand adducts, responsible for the disruption of the double helix B-conformation, thus leading to apoptotic cell death [20]–[22]. Over the last decades, several studies on the structural behavior and antineoplastic properties of Pt(II)- and Pd(II)-polyamine complexes have been carried out [3], [23]–[30]. The aim is to obtain new anticancer drugs complementary to cisplatin (used as the lead compound), with an increased activity and a reduced systemic toxicity and acquired resistance, relative to the agents presently used in the clinic (cisplatin, carboplatin and oxaliplatin). Thus, the search for structurally novel polyamine-conjugated Pt(II) and Pd(II) compounds exhibiting antineoplastic activity is of the utmost relevance in the development of improved anticancer therapeutic approaches.

In the present work, the spermidine analogue norspermidine (NSpd) and its newly synthesized trinuclear Pd(II) and Pt(II) complexes, Pd_3_NSpd_2_ (Pd-NSpd) and Pt_3_NSpd_2_ (Pt-NSpd) respectively [25], were evaluated as to their cytotoxic activity against three different human breast cancer cell lines (MCF-7, JIMT-1 and L56Br-C1) and one immortalized normal-like breast epithelial cell line (MCF-10A). NSpd is a naturally occurring triamine in some species of plants, bacteria and algae, but not in humans [31], [32], and it has shown antineoplastic activity against some types of tumors in mice [33]. It has also been shown, both *in vivo* and *in vitro*, that several triamine analogues are less toxic than the corresponding tetraamines, but their use as antitumor drugs is more favorable since they can potentially offer more therapeutic advantages [34]. The multidisciplinary study of this kind of modified polyamines and their Pd(II) and Pt(II) chelates will hopefully lead to a better understanding of the molecular basis of their biological activity, aiming at the design of improved Pd- and Pt-polyamine chemotherapeutic agents.

## Results

In this paper, we studied the effects of NSpd, Pd-NSpd or Pt-NSpd treatment on three human breast cancer cell lines (JIMT-1, L56Br-C1 and MCF-7) and one human immortalized normal-like breast epithelial line (MCF-10A).

### MTT Reduction

**Figure 1:**
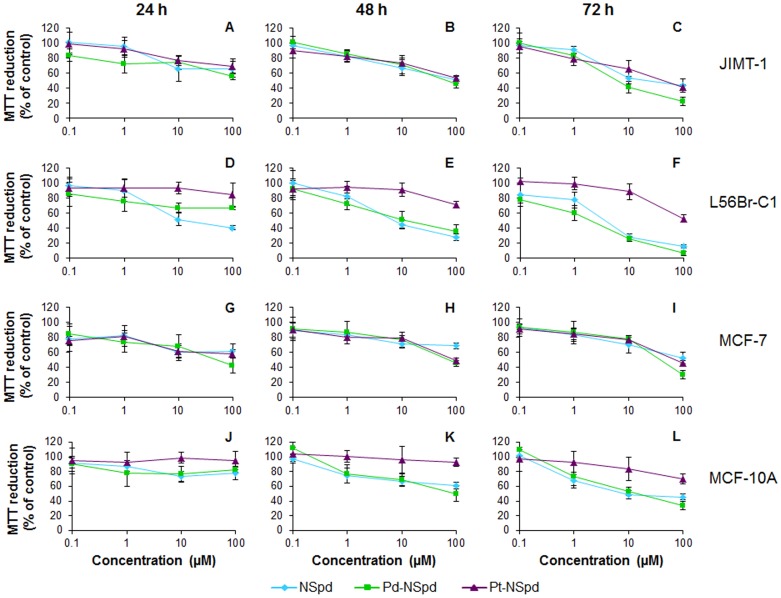
Dose response effects of NSpd, Pd-NSpd or Pt-NSpd treatment. The three breast cancer cell lines JIMT-1 (**A**–**C**), L56Br-C1 (**D**–**F**), MCF-7 (**G**–**I**) and the normal-like breast cell line MCF-10A (J–L) were used. Twenty-four h after seeding of cells in 96-well plates, the polyamine analogue and its complexes were added to the final concentrations shown in the figure and the cells were treated for 24, 48 and 72 h, before evaluation using an MTT assay. The results are expressed as % of control (n = 12 independent samples from two independent experiments) with bars representing ± SD.

In order to evaluate the toxicity of NSpd, Pd-NSpd or Pt-NSpd over a large dose range against the four cell lines investigated, a 3-(4,5-dimethyl-thiazolyl-2)-2,5 diphenyltetrazolium bromide (MTT) dose-response test that is assumed to reflect cell number was performed [35], [36]. This assay showed that MTT reduction (*e.g.* mitochondrial reductase activity) decreased in all cases when increasing both concentration and time of treatment, although the reactions to each treatment were different within the four cell lines (Fig. 1). The dose response curves show that while Pt-NSpd was found to be the least cytotoxic compound, Pd-NSpd and NSpd were more toxic and gave in general similar dose responses. The results also show that L56Br-C1 was the most sensitive cell line. The effects of drug treatment were more obvious between 10 and 100 µM concentrations, for both 48 and 72 h of treatment.

### Effect of Short and Long-time Treatment on Cell Proliferation

**Figure 2:**
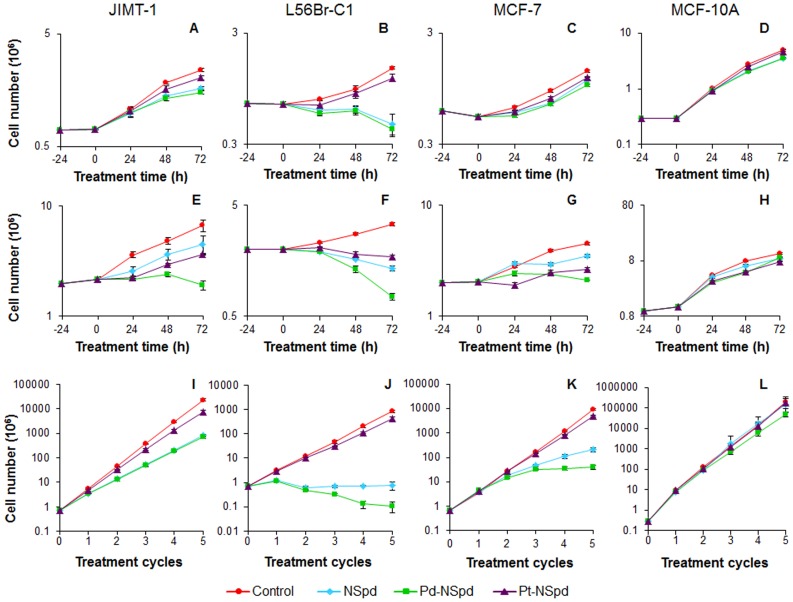
Effects of NSpd, Pd-NSpd or Pt-NSpd treatment on the proliferation of JIMT-1, L56Br-C1, MCF-7 and MCF-10A cells. Twenty-four h after seeding of cells (0 h time of treatment in the figure), NSpd, Pd-NSpd or Pt-NSpd was added to give a final concentration of 25 µM (**A**–**D**) or 100 µM (**E**–**H**). Cells were harvested by trypsinization and counted in a hemocytometer. The results are presented as mean values (n = 3−6 independent samples from one or two independent experiments) and bars represent ± SD. When not visible, the bars are covered by the symbols. **I**–**L**: Cells were seeded and NSpd, Pd-NSpd or Pt-NSpd was added to the final concentration of 25 µM after 24 h of seeding. After 72 h of treatment, the drug-containing medium was aspirated and drug free culture medium was added. After an additional 72 h of incubation, cells were harvested by trypsinization and counted in a hemocytometer. These 7 days were defined as one treatment cycle. The total recovery time between each treatment was 96 h. The cells were reseeded at the same density as at the previous passage and treated with the same drug for the next treatment cycle. All together this was repeated for 5 treatment cycles. The data are presented as the total amount of cells (mean values (n = 3−6 samples from one or two independent experiments) and bars represent ± SD) that theoretically would have accumulated if all cells had been reseeded with a known cell density after each treatment cycle. When not visible, the bars are covered by the symbols. Please note that the y-axis has different scales for the different cell lines because of different rates of cell proliferation.

Based on the dose-response curves, we decided to compare 25 and 100 µM concentrations for further investigations. We examined the effect of NSpd, Pd-NSpd or Pt-NSpd treatment on cell proliferation using both concentrations. The proliferation of MCF-10A cells (Fig. 2) was only slightly affected by treatment with a 25 µM concentration of NSpd, Pd-NSpd or Pt-NSpd, while in JIMT-1 and MCF-7 cells the proliferation was more affected than in MCF-10A cells and differential effects of the drugs were clearly discernible (Fig. 2). L56Br-C1 cells were the most sensitive to these compounds and there was even a decrease in cell number implicating cell death after 72 h of treatment with NSpd and Pd-NSpd (Fig. 2). Figure S1 shows the morphology of the cells after 72 h of treatment with the compounds. L56Br-C1 cells had to be rinsed with PBS to remove all dead floating cells before photography of attached cells that were found in small patches.

For the 100 µM concentration, more distinct differences were obtained between the tested compounds and the cell lines than for treatment with a 25 µM concentration (Fig. 2 **E**–**H**). MCF-10A cells were still the least sensitive with smallest differences between the different treatments (Fig. 2). Pd-NSpd treatment appeared to inhibit cell proliferation in JIMT-1 and MCF-7 cells almost immediately, while Pt-NSpd and NSpd slowed cell proliferation, with the former being slightly more efficient than the later (Fig. 2 **E,G**). In L56Br-C1 cells, treatment with 100 µM NSpd, Pd-NSpd or Pt-NSpd immediately inhibited cell proliferation and a marked reduction in cell number was observed in Pd-NSpd-treated cultures (Fig. 2 **F**), implicating cell death.

We also investigated the effect of repeated cycles of 72 h treatment with 25 µM NSpd, Pd-NSpd or Pt-NSpd followed by 96 h of drug withdrawal on cell proliferation (Fig. 2 **I**–**L**). MCF-10A normal-like breast cells were not affected by repeated treatment cycles with either NSpd or Pt-NSpd and were slightly affected by Pd-NSpd (Fig. 2). In L56Br-C1 cells, repeated treatment cycles with NSpd or Pd-NSpd resulted in unchanged and decreasing cell numbers, respectively, compared to seeding at time 0, while repeated treatment with Pt-NSpd only slightly affected the cell number compared to control (Fig. 2). In JIMT-1 and MCF-7 cells, the accumulated cell number was lower for cells treated with either NSpd or Pd-NSpd, but not with Pt-NSpd, when compared with untreated cells, especially after the second treatment cycle (Fig. 2).

### Competition with ^3^H-spermidine in Uptake

**Figure 3:**
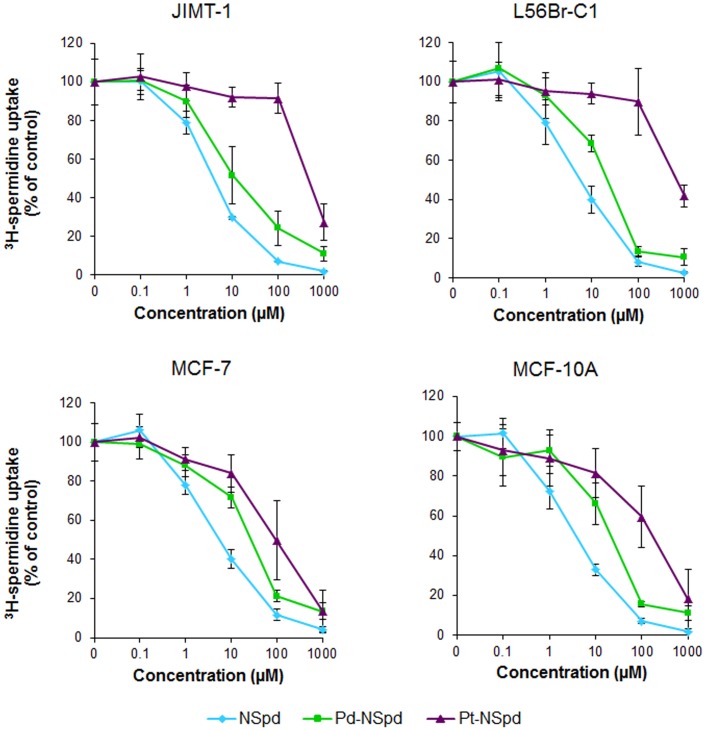
Effects of NSpd, Pd-NSpd or Pt-NSpd on the uptake of ^3^H-spermidine in JIMT-1, L56Br-C1, MCF-7 and MCF-10A cells. The cells were seeded in 12 well plates and incubated for 48 h, whereupon the polyamine analogue or its complexes were added to give the final concentrations shown in the figure. The concentration of ^3^H-spermidine used was 1 µM. The results are presented as mean values (n = 4 samples from two independent experiments) and bars represent ± SD.

This assay was used to evaluate the ability of NSpd, Pd-NSpd or Pt-NSpd to competitively inhibit the uptake of ^3^H-spermidine (^3^H-Spd) in the four breast cell lines. For this purpose, five different concentrations were tested. Fig. 3 shows that, in all cell lines, only NSpd clearly competed with ^3^H-Spd, resulting in a 50% inhibition of ^3^H-Spd uptake at a 6–8 µM NSpd concentration. The concentration needed for 50% inhibition of ^3^H-Spd uptake was approximately 100 times higher for Pt-NSpd than for NSpd in the four cell lines. Pd-NSpd, contrary to Pt-NSpd, resulted in a 50% inhibition of ^3^H-Spd uptake at concentrations that were only twice as high as NSpd in JIMT-1 cells (about 15 µM) and between five and six times higher in the other three cell lines.

### ODC Activity

**Figure 4:**
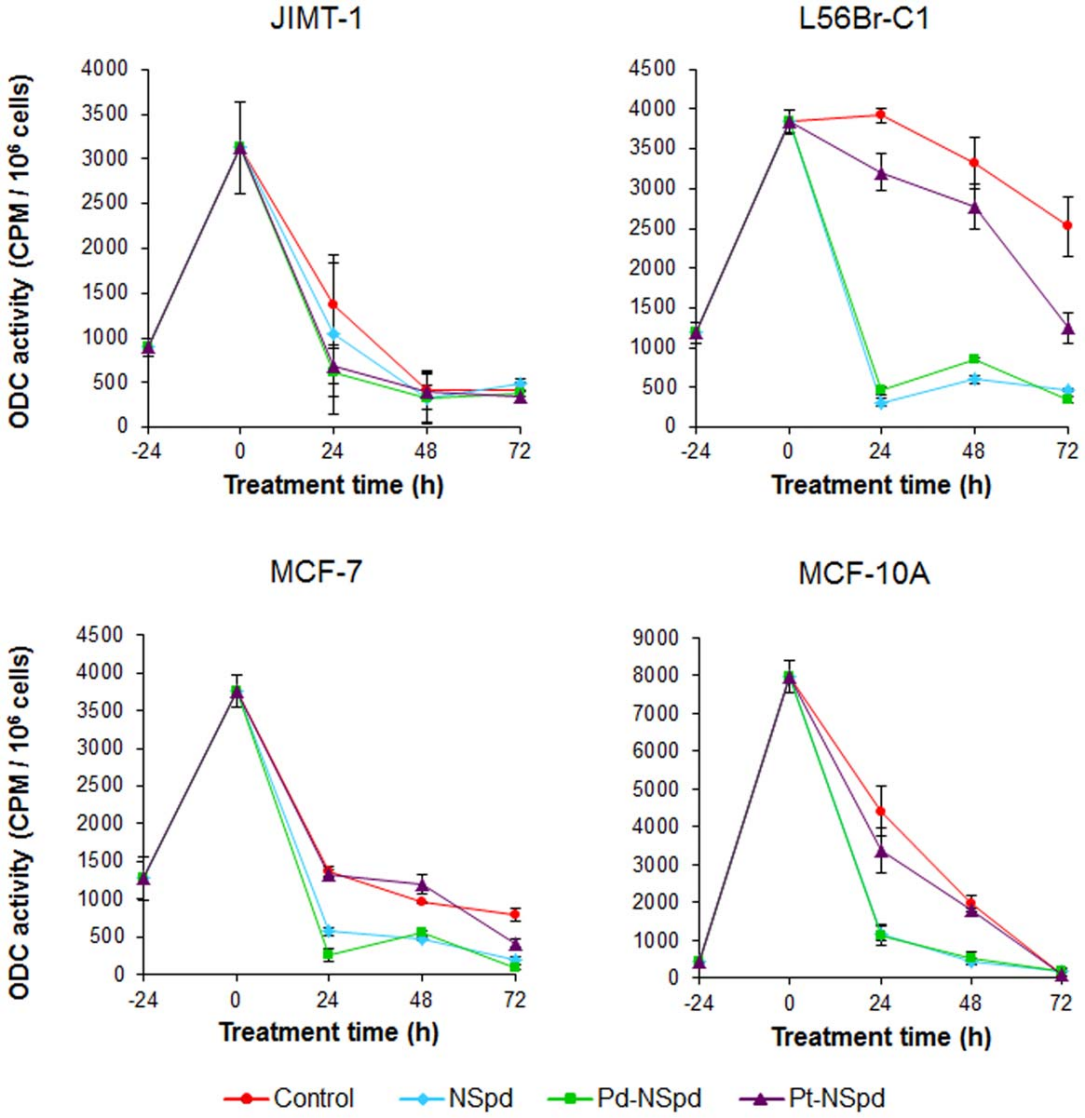
ODC activity in JIMT-1, L56Br-C1, MCF-7 and MCF-10A cells treated with NSpd, Pd-NSpd or Pt-NSpd. Twenty-four h after seeding of cells (0 h time of treatment in the figure), NSpd, Pd-NSpd or Pt-NSpd was added to give a final concentration of 25 µM. The ODC activity was determined using a radiometric assay. The results are presented as mean values (n = 3 independent samples from one independent experiment) and bars represent ± SD.

In all cell lines, there was an evident rise in ornithine decarboxylase (ODC) activity during the first day after seeding (Fig. 4). Thereafter, ODC activity decreased rapidly in all cell lines except in L56Br-C1 cells, where it remained elevated for at least another day before slowly decreasing. Treatment with NSpd or Pd-NSpd clearly decreased the ODC activity in all the cell lines examined, except for the JIMT-1 cells where only minor differences were seen (Fig. 4). The effect on ODC activity was most evident in L56Br-C1 cells, as the ODC activity remained relatively high in the control cells for the entire experimental period. Pt-NSpd only slightly inhibited the ODC activity in all cell lines analyzed.

### Cell Cycle Phase Distribution and Cell Death

Flow cytometry (FCM) represents a fast method to determine the cell cycle phase distribution based on the stoichiometric binding of a fluorescent probe to DNA. As the treatments were shown to affect cell proliferation, we further examined whether there were changes in the cell cycle phase distribution induced by each compound and whether they induced cell death (studied by monitoring the appearance of a sub-G_1_ peak) using FCM. Only data from treatment with 100 µM concentration are shown, as the pattern of changes was similar with 25 µM concentration, but less pronounced. Representative DNA histograms obtained after 72 h of treatment are shown as Figure S2.

**Figure 5:**
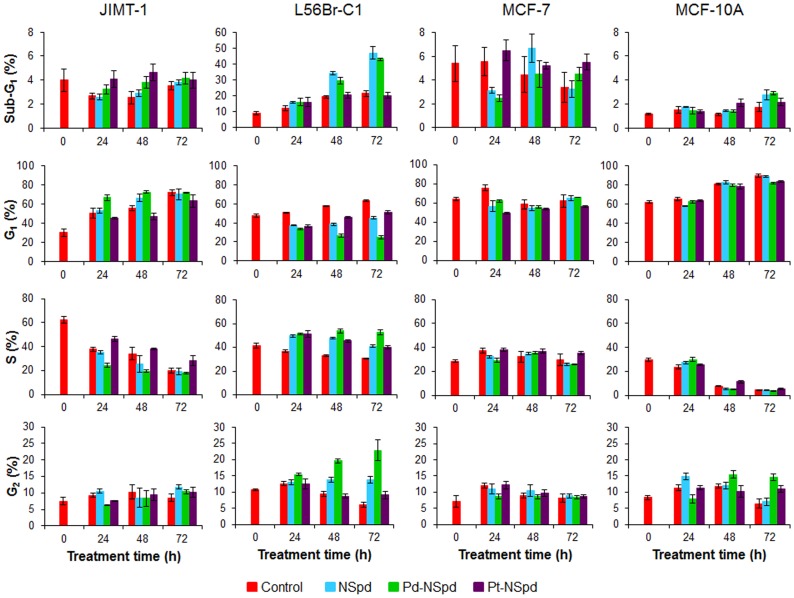
Sub-G_1_ region and cell cycle phase distribution of JIMT-1, L56Br-C1, MCF-7 and MCF-10A cells treated with NSpd, Pd-NSpd or Pt-NSpd. Twenty-four h after seeding the cells, NSpd, Pd-NSpd or Pt-NSpd were added to give a final concentration of 100 µM. At 24, 48 and 72 h of treatment, both detached and attached cells were harvested, pooled and fixed in 70% ice-cold ethanol. The nuclei were stained with propidium iodide and the analysis was performed using flow cytometry. The results are presented as mean values (n = 3−6 independent samples from one or two independent experiments) and bars represent ± SD.

The data show that the only cell line in which the percentage of cells in the sub-G_1_ region increased substantially was the L56Br-C1 cell line (Fig. 5). When treating L56Br-C1 cells with NSpd or Pd-NSpd, cell death was induced after 48 h of treatment as indicated by the increased sub-G_1_ region and it further increased after 72 h of treatment. No cell death was observed in the other cell lines analyzed.

The most obvious changes in cell cycle phase distribution were found in JIMT-1 and L56Br-C1 cells, while there were no clear changes compared to control in MCF-7 and MCF-10A cells (Fig. 5). In JIMT-1 cells, the percentage of cells in the G_1_ phase increased from 30 to almost 70% during the first 24 h of treatment with 100 µM Pd-NSpd and then remained at that level. During the same time period, the S phase decreased from 60 to 20%. Less evident differences in cell cycle phase distribution were observed in NSpd and Pt-NSpd-treated JIMT-1 cells. In L56Br-C1, the G_1_ phase decreased in Pd-NSpd-treated cells, while both S and G_2_ phases increased. NSpd and Pt-NSpd treatments resulted in similar changes in cell cycle phase distribution in L56Br-C1 cells and they were largely in between those of control and Pd-NSpd.

### Cell Cycle Kinetics

Since we found effects on the cell cycle phase distribution and induced cell death in L56Br-C1 cells, it was of interest to further study cell cycle kinetics. For this purpose, the length of both S and G_2_+M phases were evaluated by the use of a bromodeoxyuridine (BrdUrd)-FCM method [37], [38].

The length of the S phase corresponds to the DNA replication time and was calculated by following the movement of BrdUrd-labelled cells during the cell cycle according to the principles of Begg *et al* [39] as previously described [38], [40]. The S phase was significantly prolonged in all cell lines after 72 h of NSpd or Pd-NSpd treatment, compared to control cells (Table 1). However, the prolongation was different in the four cell lines. Pt-NSpd treatment resulted in the smallest increase in S phase length and no increase in MCF-10A cells.

**Table 1:** Treatment with NSpd, Pd-NSpd or Pt-NSpd affected the lengths of the S and G2+M phases1.

S phase length (h)								
Cell Line	Control		NSpd			Pd-NSpd		Pt-NSpd
JIMT-1	13.1±1.0		21.4±0.9***			20.2±1.3***		14.1±1.0
L56Br-C1	15.7±1.0		18.5±1.6***			22.5±1.3***		16.7±0.5*
MCF-7	11.8±1.1		15.2±2.4**			14.8±1.7**		13.3±1.4*
MCF-10A	7.6±0.3		8.7±1.1*			15.7±1.1***		7.1±0.1
**G_2_+M phase length (h)**								
**Cell Line**		**Control**		**NSpd**	**Pd-NSpd**		**Pt-NSpd**	
JIMT-1		4.5		5.7	5.7		5.1	
L56Br-C1		5.8		7.5	7.5		7.2	
MCF-7		4.2		5.1	5.3		4.9	
MCF-10A		3.7		6.2	5.8		4.3	

1 Cells were seeded and the drugs (25 µM) were added 24 h later. After 72 h of treatment, the cells were labelled with bromodeoxyuridine (BrdUrd) for 30 minutes, before the cells were allowed to progress through the cell cycle in BrdUrd free medium. Cells were sampled for analysis of DNA and BrdUrd contents by flow cytometry at 3, 6, 9, and 12 h post-labelling. Data was collected from one experiment, n = 5−12.

* p<0.05;

** p<0.01;

*** p<0.001.

The length of G_2_+M phase was also evaluated and corresponds to the time required for cells to proceed through the G_2_ and M phases of the cell cycle [41]. The data indicated a prolongation of the G_2_+ M phase in all four cell lines after 72 h of treatment with NSpd, Pd-Npd or Pt-NSpd, compared to control cells.

### Effects of NSpd, Pd-NSpd or Pt-NSpd Treatment on Colony Forming Efficiency

This assay is designed to measure the ability of cells to proliferate and form colonies in an anchorage independent manner and it can be used to assess the sensitivity of human tumors to anticancer drugs. The normal-like cell line MCF-10A does not form colonies in soft agar and was not used in this assay. In the breast cancer cell lines, the colony forming efficiency decreased by all treatments compared to the control, with Pd-NSpd being the most effective compound and Pt-NSpd the least effective one (Table 2).

**Table 2:** NSpd, Pd-NSpd or Pt-NSpd treatments of JIMT-1, L56Br-C1 and MCF-7 cells reduced the colony forming efficiency in soft agar^1^.

Cell line	JIMT-1	L56Br-C1	MCF-7
**Control (%)**	24.2±2.1	30.7±2.3	28.3±2.7
**NSpd (%)**	14.2±3.1 (58.5)	14.1±2.6 (46)	18.5±2.1 (65.5)
**Pd-NSpd (%)**	12.3±2.4 (50.8)	12.1±2.7 (39.4)	15.8±2.4 (55.9)
**Pt-NSpd (%)**	19.3±2.8 (79.8)	24.0±1.3 (78.1)	24.2±1.7 (85.5)

1 Cells were seeded and the drugs (25 µM) were added 24 h later. After 72 h of treatment, the cells were harvested, counted and reseeded at low density in soft agar. Colonies were counted after 14 days of incubation. The results are presented as mean values (n = 3 independent cultures) ± SD and as percentage of control (in brackets).

### Intracellular Pd and Pt Accumulation

**Figure 6:**
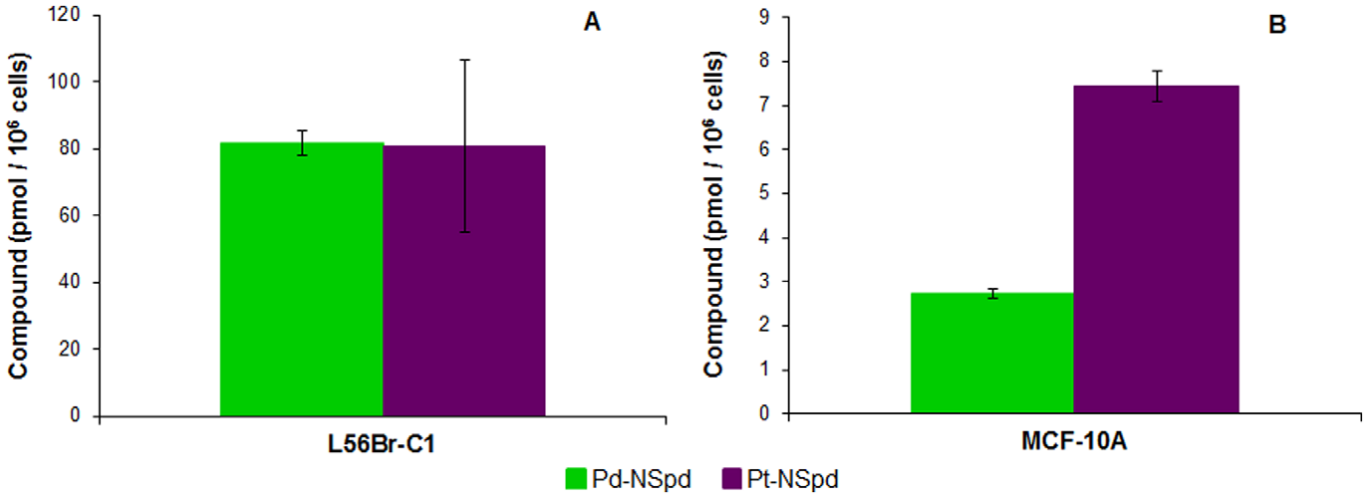
Intracellular accumulation of Pd-NSpd and Pt-NSpd in L56Br-C1 and MCF-10A cells. At 72 h of treatment with 25 µM of Pd-NSpd and Pt-NSpd, cells were harvested, pooled and digested in HNO_3_. The supernatant was used for analysis of Pd and Pt by ICP-MS and the data used to calculate the intracellular Pd-NSpd and Pt-NSpd concentrations. The results are presented as mean values (n = 3 independent samples from one independent experiment) and bars represent ± SD.

The cellular contents of Pd- and Pt-complexes after treatment with 25 µM Pd-NSpd and Pt-NSpd, respectively, for 72 h were estimated by measuring the amount of Pd and Pt in the cells using inductively coupled plasma mass spectrometry (ICP-MS). As shown in Fig. 6, the intracellular concentrations of Pd-NSpd and Pt-NSpd were considerably higher in the most sensitive cell line, L56Br-C1 compared to the least sensitive one, MCF-10A. Moreover, the concentration of Pt-NSpd was 2–3 times as high as that of Pd-NSpd in MCF-10A cells, whereas in L56Br-C1 cells the concentrations were similar.

### Assessment of Genotoxic Potential of NSpd, Pd-NSpd or Pt-NSpd using Anthem’s Genotox Screen

**Figure 7:**
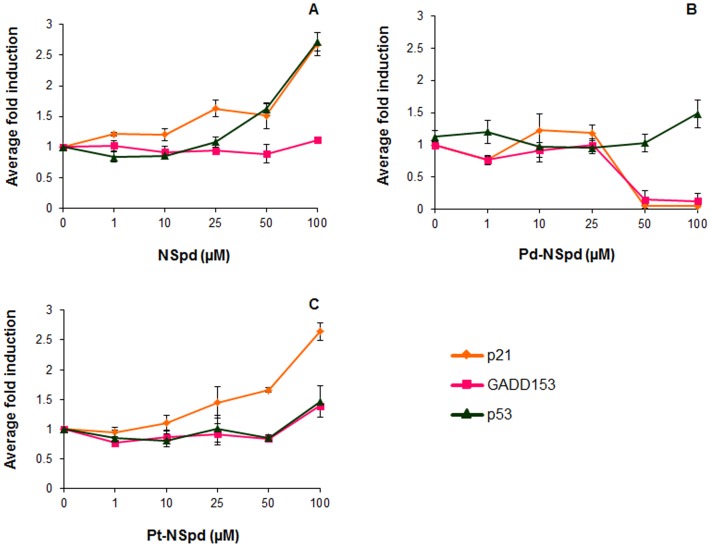
Genotoxic effects of NSpd, Pd-NSpd or Pt-NSpd in genetically engineered reported-based HCT-p21-GADD-p53 cells. The genetically engineered reporter-based HCT-p21-GADD-p53 cells carrying the DNA damage early sensors p21, GADD153 and p53 were seeded in 96-well plates and allowed to attach for 16 h. The p21 promotor was operatively linked to Renilla luciferase reporter gene, the GADD 153 promotor operatively linked to firefly luciferase reporter gene and the p53 response elements operatively linked to β-galactosidase reporter gene. Sixteen h after seeding, the polyamine analogue and its complexes were added to the final concentrations shown in the figure and incubated for 72 h. The samples were sampled and analyzed for Renilla luciferase, firefly luciferase and β-galactosidase. The results are presented as mean values (n = 3 independent samples from one independent experiment) and bars represent ± SD.

Anthem’s Genotox screen is a patented human cell-based technology that employs an engineered stable p53 proficient HCT116 cell line. This mammalian genotoxicity screen can help in the identification of potential genotoxins in an early phase of drug development. Therefore, the goal of this assay was to detect if the test compounds were potential genotoxins in this screen, as a complement to the single cell gel electrophoresis assay. A test compound is considered to be genotoxic if the average fold reporter gene induction at any test dose is over 1.5 fold. Taking this into account, Fig. 7 shows that 100 µM NSpd induced both p21 and p53, Pt-NSpd induced only p21 at the test concentrations of 50 and 100 µM and Pd-NSpd did not induce either of the early DNA damage sensors (p21, GADD153 and p53) at any concentration in the Anthem’s Genotox screen. Therefore, NSpd and Pt-NSpd were found to be potential genotoxins, but not Pd-NSpd.

### Single Cell Gel Electrophoresis

**Figure 8:**
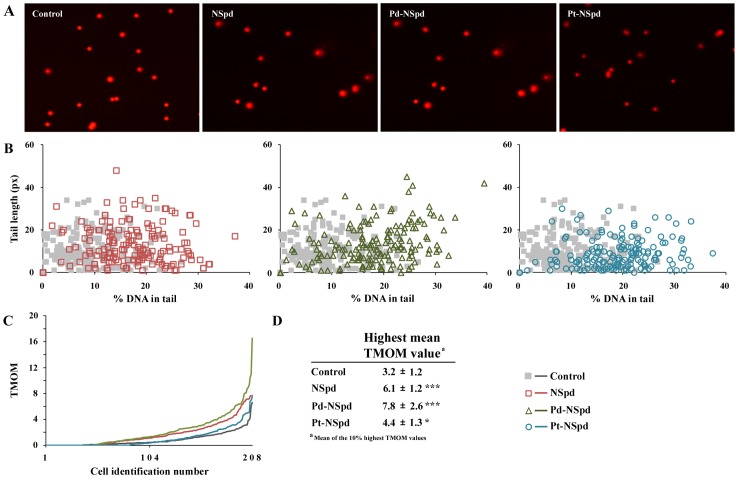
The single cell gel electrophoresis (SCGE) assay was used to evaluate DNA damage in L56Br-C1 cells. Twenty-four h after seeding of L56Br-C1 cells, NSpd, Pd-NSpd or Pt-NSpd was added to give a final concentration of 25 µM. After 72 h of treatment, cells were harvested for SCGE analysis. The ethidium bromide-stained nucleoids were photographed and then examined using the Comet Score™ Freeware. **A**. Images of comets obtained by the SCGE assay. DNA damage results in comets with head and tail, whereas undamaged DNA results in a round head. **B**. Percentage DNA in tail on the x-axis *versus* tail length on the y-axis for individual cells. **C**. Tail moment TMOM (%DNA in tail multiplied by tail length) for individual cells. Data were collected from three independent experiments, n = 207 cells. **D**. Table showing the mean TMOM value of the 10% highest TMOM values *i.e.* 20 highest values ± SD. *p<0.05 compared to control; ***p<0.001 compared to control.

The single cell gel electrophoresis (SCGE) assay was performed to investigate if the compounds induced DNA strand breaks. None of the compounds induced any significant amount of DNA strand breaks with a 25 µM concentration in any of the cell lines tested. Only data for the most sensitive cell line (L56Br-C1) are shown (Fig. 8). Fig. 8 **A** shows representative comets from the different treatments. The tail length and the % DNA in the tail was evaluated for each comet and plotted in cytograms (Fig. 8 **B**). TMOM for each cell was calculated and plotted (Fig. 8 **C**). Fig. 8 **D** shows a table with the mean TMOM value of the 10% highest TMOM values. Corresponding data for JIMT-1 and MCF-10A cells are shown in Figures S3 and S4, respectively.

## Discussion

Breast cancer is a complex disease and it affects, approximately, one in ten women from North Western Europe and America, out of which 1/3 die of their disease [1]. For this reason, new treatment strategies are needed and the initial step is to test new chemotherapeutic drugs in breast cancer cell lines, to choose candidates for further studies towards clinical use.

Cisplatin has a central role in cancer chemotherapy and is used for the treatment of various forms of cancer. However, it exerts severe side effects and leads to acquired resistance, which limits its clinical use [21]. For this reason, it is of great importance to test new metal-based antitumor agents, particularly Pd(II) and Pt(II) chelates, which can lead to a higher efficacy and specificity regarding DNA binding. In fact, although the initial concept relied on the fact that Pd(II) compounds were biologically inactive as antineoplastic agents due to the high lability of the Pd(II) centre, it has lately been shown that in some cases they are not only more active than cisplatin, but also more efficient than their analogous Pt(II) complexes [29]. Thus, new Pd- and Pt-based compounds have been synthesized and tested as potential anticancer agents, namely Pd(II) and Pt(II) polynuclear complexes with polyamines [28], [29], [42], [43].

Polyamines play important roles in a variety of processes, including cell proliferation, thus their metabolic pathway represents a potential target for cancer intervention and can be manipulated by either inhibiting polyamine biosynthesis and uptake and/or stimulating polyamine catabolism. Polyamine analogues have been shown to lead to polyamine depletion, by affecting the biosynthetic as well as the catabolic processes, resulting in inhibition of cell proliferation and induction of cell death, rendering them promising agents against cancer [35].

In the present study we investigated the cytotoxic effects of the spermidine analogue NSpd and its newly synthesized trinuclear Pd(II) and Pt(II) complexes (Pd-NSpd and Pt-NSpd, respectively) in three breast cancer cell lines (JIMT-1, L56Br-C1 and MCF-7) and one immortalized normal-like breast epithelial cell line (MCF-10A). The results showed that the different cell lines reacted differently to the treatment with NSpd, Pd-NSpd or Pt-NSpd, the normal-like MCF-10A cell line being the least sensitive.

As determined by the MTT assay, administration with NSpd, Pd-NSpd or Pt-NSpd resulted in decreased cell viability and increased growth inhibition in a dose- and time-dependent manner. MCF-10A was shown to be the least affected cell line while L56Br-C1 was the most sensitive one. Interestingly, the polyamine analogue NSpd, which has not been tested before, presented a clear growth inhibitory effect. The effect obtained with the Pd(II) complex of NSpd was usually similar to that obtained with NSpd. However, a clear difference was seen between the two NSpd complexes. In fact, Pd-NSpd treatment resulted in much higher MTT reduction than Pt-NSpd treatment in all the cell lines tested, demonstrating it to be considerably more cytotoxic than the homologous Pt(II) complex.

Similar results were obtained in a cell proliferation assay in which the L56Br-C1 was the most sensitive cell line, whereas the MCF-10A cells were hardly affected by either of the drugs. In this assay, Pd-NSpd was shown to have a somewhat stronger antiproliferative effect on the breast cancer cell lines than NSpd. In addition, the Pd-NSpd effect was also stronger than that of the homologous Pt(II) complex. Results from repeated cycles of drug treatment and withdrawal demonstrated that the breast cancer cells did not easily recover from the treatment with NSpd or Pd-NSpd. Again, the L56Br-C1 cells were the most sensitive ones, demonstrating an unchanged or decreased cell number after repeated cycles of treatment with NSpd or Pd-NSpd, respectively. Interestingly, the breast cancer cells appeared to recover quickly from the antiproliferative effect of Pt-NSpd. Only minor effects on cell proliferation were observed when treatment with Pt-NSpd was combined with withdrawal periods. The results are in accordance with published studies regarding other Pd or Pt-polyamine complexes, namely Pd(II) and Pt(II)-spermine complexes, where the substitution of Pd(II) for Pt(II) increased the cytotoxicity of the drug [43].

In most cell types, two classes of polyamine transport systems have been recognized: one that is sodium dependent with a preference for putrescine and one that is sodium independent with a preference for spermidine and spermine. The polyamine transport system is energy-, time-, temperature-, and concentration-dependent and saturable, suggesting it is a carrier-mediated transport. However, this system has a low specificity and, thus, can be responsible for the transport of diverse molecules, like polyamine-based compounds, into the cell [7], [44]. In general, and since the transporter is not specific for putrescine, spermidine or spermine, the affinity of the carrier increases to amines which have chain lengths resembling those of spermidine or spermine. It has also been shown that the primary nitrogen groups from the polyamines seem to be critical for uptake [45]. Primary amines are present in the linear NSpd, Pd-NSpd and Pt-NSpd (terminal amino groups), thus pointing to a possibility of these compounds being taken up by the polyamine transporter. In this study, we showed that only NSpd competed efficiently with ^3^H-Spd uptake in all four cell lines, indicating that NSpd uses the same polyamine transport system as spermidine to enter the cell. Regarding the complexes, Pd-NSpd inhibited ^3^H-Spd uptake to a higher degree compared to Pt-NSpd, but still much less compared to NSpd. It is unclear how these chelates are transported into the cell but, since they share similarities with cisplatin, it is possible that they enter the cell in the same way. For many years the general belief was that cisplatin entered the cells by passive diffusion across the plasma membrane, but recently a transmembrane protein involved in copper homeostasis (copper transporter 1, CTR1) was found to modulate the uptake of Pt(II)-based anticancer drugs, such as cisplatin. Currently, the CTR1 is known to act as a major platinum-drug transporter in several cell systems [46]. Therefore, the presently investigated complexes might enter the cell using the CTR1 transporter or, since they are considerably more lipophilic than cisplatin (due to the presence of the alkylpolyamine ligands), by passive diffusion across the membrane, possibly by partly using the polyamine transporter system. Again, minor structural differences can have important effects on activity, which was clearly shown through the substitution of a Pd by a Pt center [28], [43].

The differences in cytotoxicity observed between Pd-NSpd and Pt-NSpd complexes may be caused by differences in cellular uptake. Attempts to estimate the content of the complexes in L56Br-C1 and MCF-10A cells revealed a large difference between the two cell lines. The most sensitive cell line, L56Br-C1, contained more than 10-fold higher concentrations of Pd-NSpd or Pt-NSpd compared to the least sensitive cell line MCF-10A, after treatment with the complexes for 3 days. The reason for this difference is not known. No major differences were seen in the ability of the complexes to inhibit the ^3^H-Spd uptake in the cells, indicating that the uptake of the complexes may be unrelated to the polyamine transport system. Interestingly, the data showed that in MCF-10A cells the content of Pt-NSpd was higher than of Pd-NSpd after treatment with the complexes, suggesting a more efficient uptake of Pt-NSpd than of Pd-NSpd. A similar difference was also observed in JIMT-1 cells (results not shown), but not in the L56Br-C1 cells, which contained equal amounts of the complexes after treatment. Nevertheless, Pd-NSpd exhibited stronger cytotoxicity than Pt-NSpd in all of the breast cancer cell lines tested, making it a more efficient agent from a cancer treatment approach.

High expression of ODC characterizes some cancers, including breast cancer. Consequently, there has been a great effort to search for new compounds that can inhibit ODC activity in tumor cells [47]. The increase in ODC activity in all cell lines registered 24 h after seeding is correlated to cell proliferation. The reason for ODC activity remaining elevated for somewhat longer time in L56Br-C1 cells, compared to the other cell lines, is not known. However, this cell line has the slowest growth rate of the ones used in the present study and it is conceivable that the duration of the ODC peak is somewhat connected to the growth rate of the cells. NSpd or Pd-NSpd treatment resulted in ODC inhibition in all the cell lines, except for the JIMT-1 cells. In comparison, no significant inhibition was observed with Pt-NSpd treatment. Although the ODC activity was significantly suppressed by NSpd or Pd-NSpd treatment in L56Br-C1, MCF-7 and MCF-10A cells, it is not clear if it contributes to the compounds cytotoxicity. Overall, there did not seem to be a clear correlation between the effects of the drugs on ODC activity and their antiproliferative effects (*i.e.* compare growth inhibitory effects and ODC suppression in JIMT-1 and MCF-10A cells). However, polyamine analogues are known to also activate the polyamine catabolic pathway leading to the depletion of the biogenic polyamines, which may be part of the growth inhibitory mechanism.

The DNA distribution of a cell population evaluated by FCM gives information about cell cycle phase distribution and cell death. The most consistent changes in cell cycle phase distribution were found in L56Br-C1 and JIMT-1 cells. Treatment of L56Br-C1 cells with Pd-NSpd or NSpd resulted in an increase in the sub-G_1_ region. This increase of the sub-G_1_ region was substantially higher after 72 h of treatment, demonstrating that treatment with either Pd-NSpd or NSpd induced cell death in this cell line. Consequently, the whole cell cycle phase distribution was affected, resulting in a decrease in the G_1_ phase along with an increase in both S and G_2_ phases. Since cell death was induced and no cell proliferation was observed, cell death presumably took place in G_1_ cells. There was no marked cell death observed in JIMT-1, MCF-7 and MCF-10A cells by either of the drugs. However, in JIMT-1 cells the G_1_ phase was increased already after 24 h of treatment with Pd-NSpd, indicating a block in this phase. This is also obvious from the growth curve analysis where we detected a block in cell proliferation at the same time point. In accordance with the results from the other growth experiments, no clear differences in cell cycle phase distribution were observed in MCF-10A and MCF-7 cells treated with NSpd, Pd-NSpd or Pt-NSpd. Nevertheless, as small effects on the cell cycle progression may be highly significant from a pharmacological point of view, it was important to fully evaluate the cell cycle progression, namely the length of S and G_2_+M phases, by the use of a DNA bromodeoxyuridine (BrdUrd) flow cytometric method. Compared to controls, NSpd and Pd-NSpd-treated cells showed a significant prolongation of the S phase as well as the G_2_+M phase in all cell lines. The prolongation of both phases was less evident in Pt-NSpd-treated cells. Polyamines are known to stabilize DNA, so replacement of natural polyamines with analogues may result in DNA destabilization that could have caused the S phase prolongation in NSpd-treated cells. On the other hand, the interaction of the chelates with DNA occurs by covalent linkage to the nitrogen atoms of the bases, mainly the N_7_ of guanine and adenine, therefore disrupting the base-paring. These complexes form long-range, intra- and inter-strand DNA adducts that are responsible for a more severe and less reparable damage than their counterparts (for instance, cisplatin) [48]. Effects on cell cycle kinetics are often related to cell cycle regulatory proteins and polyamine levels. However, in this study we did not analyze any cell cycle regulatory protein nor polyamine pools and thus no further conclusions can be drawn.

The results are further emphasized by the colony forming efficiency in soft agar. The ability of forming colonies in soft agar is characteristic of malignant cells and reproduces the invasiveness of the cancer, so only the breast cancer cell lines were analyzed. This assay showed a decrease in colony formation in all treated cells compared to the control, after 72 h of treatment. Again, this reduction was higher in NSpd or Pd-NSpd-treated cultures. The largest effect was obtained in the L56Br-C1 cell line with a decrease in colony forming efficiency of over 50%.

In the mammalian genotoxicity screen NSpd and Pt-NSpd were found to be potential genotoxins at 50 µM and 100 µM concentrations. In contrast, Pd-NSpd did not show any genotoxicity at any of the concentrations tested in the mammalian genotoxicity screen, however, we cannot rule out genotoxicity in other assays. In agreement with these results, the single cell gel electrophoresis assay did not shown any induction of DNA strand breaks with 25 µM NSpd, Pd-NSpd or Pt-NSpd treatment in any of the cell lines. However, taking these results together, we can expect that induction of DNA strand breaks could be detectable when using a higher concentration of the drug (50 µM for NSpd and 100 µM for Pt-NSpd).

In conclusion, the present results show that NSpd, Pd-NSpd or Pt-NSpd treatment of human breast cancer cells induced a variety of responses. Different cell lines showed different reactions to the drugs, L56Br-C1 being the most sensitive. The overall data in the present study demonstrates that the Pd-NSpd complex displayed stronger antiproliferative effects on the human breast cancer cell lines tested, as compared to Pt-NSpd. Similar findings were observed when comparing the cytotoxic effects of trinuclear Pd(II) chelates of spermidine and spermine to those of their Pt(II) analogues [26]–[29]. Actually, substituting Pt(II) for Pd(II) was shown to significantly increase the antineoplastic effect against the human squamous carcinoma cell line (HSC-3) [27], [28]. The reason for this cytotoxicity enhancement is likely to be related to a more efficient interaction with DNA, yielding interstrand long-range adducts less prone to repair, and hence a more severe damage. Furthermore, this seems to be a selective effect, since the cytotoxicity activity of the Pd(II) and Pt(II) polyamine complexes presently studied varied depending on the cell line [26]–[29]. Interestingly, epithelial-derived cancer cells were more sensitive to the spermidine Pt(II) complexes than the leukemic cell lines [27]. In general, cancer cells seemed more sensitive than the nonneoplastic ones [27], [29]. Nevertheless, further investigations are needed in order to achieve a more detailed analysis of the antitumor potential of Pd(II) and Pt(II) complexes with this kind of linear polyamines.

## Methods

### Chemicals

Cell culture medium components were purchased from Biochrom, Berlin, Germany. Tissue culture plastics were acquired from Nunc, Roskilde, Denmark. Phosphate-buffered saline (PBS: 8 g/L NaCl, 0.2 g/L KCl, 1.15 g/L Na_2_HPO_4_, 0.2 g/L KH_2_PO_4_, pH 7.3) was purchased from Oxoid Ltd., Basingstoke, Hampshire, UK. Nonidet P-40 was purchased from VWR, Lund, Sweden. Insulin, hydrocortisone, propidium iodide (PI), aminoguanidine, 3-(4,5-Dimethyl-thiazolyl-2)-2,5 diphenyltetrazolium bromide (MTT) and poly(2-hydroxyethyl methacrylate) (polyHEMA) were obtained from Sigma, Stockholm, Sweden. Epithelial growth factor (EGF) was purchased from Invitrogen AB, Stockholm, Sweden. Dimethyl sulphoxide (DMSO) was acquired from Merck KGaA, Darmstadt, Germany. L-[1-^14^C]Ornithine (52 mCi/mmol) was purchased from New England Nuclear Du Pont, Scandinavia AB, Stockholm, Sweden. Nusieve® GTG low-melting-point agarose was obtained from FMC BioProducts, Rockland, ME, USA. Bromodeoxyuridine (BrdUrd) was obtained from DAKO Cytomation Denmark A/S, Glostrup, Denmark. Primary monoclonal antibody against BrdUrd (M744) and secondary fluorescein isothiocyanate antibody (F313) were purchased from Dakopatts, Glostrup, Denmark. The spermidine trihydrochloride [terminal methylenes –^3^H (N)] (32.4 Ci/mmol) was acquired from PerkinElmer, Boston, MA, USA. The polyamine analogue norspermidine trihydrochloride (NSpd) (98%) was purchased from Sigma-Aldrich Chemical Co, Sintra, Portugal. Its Pd-NSpd and Pt-NSpd complexes were synthesized as previously described [25].

### Cell Lines and Cell Culture

One normal-like breast epithelial cell line MCF-10A [49] and three breast cancer cell lines (MCF-7, JIMT-1 and L56Br-C1) [50] were used in the study. MCF-10A is an immortalized, non-transformed epithelial cell line derived from human fibrocystic mammary tissue that has retained many normal traits as anchorage dependence and thus, cannot form colonies in soft agar. The three breast cancer cell lines have different partly known genetic defects and belong to different breast cancer sub-groups. MCF-7 belongs to the luminal A sub-group. JIMT-1 belongs to the HER-2 breast cancer sub-group while L56Br-C1 belongs to the basal breast cancer sub-group.

The L56Br-C1 cell line was established at the Department of Oncology, Clinical Sciences, Lund University, Sweden [51]. The JIMT-1 cell line was purchased from the German Collection of Microorganisms and Cell Cultures, DSMZ (Braunschweig, Germany) and the MCF-7 and MCF-10A cell lines were obtained from the American Tissue Type Culture Collection (Manassas, VA, USA). The cell lines were cultured as monolayers at 37°C in a humidified incubator with 5% CO_2_ in air. MCF-7 cells were maintained in RPMI 1640 medium supplemented with 10% fetal calf serum (FCS), non-essential aminoacids (1 mM), insulin (10 µg/ml), penicillin (100 U/ml) and streptomycin (100 µg/ml). L56Br-C1 cells were cultured in the medium with the same composition as that for MCF-7 cells, but with heat-inactivated FCS and the addition of sodium-pyruvate (1 mM). JIMT-1 cells were cultured in DMEM/Ham’s F12 medium supplemented with 10% FCS, non-essential aminoacids (1 mM), insulin (10 µg/ml), penicillin (100 U/ml) and streptomycin (100 µg/ml). MCF-10A cells were maintained in RPMI 1640 medium supplemented with 10% heat-inactivated FCS, non-esssential aminoacids (1 mM), insulin (10 µg/ml), penicillin (100 U/ml), streptomycin (100 µg/ml), epidermal growth factor (20 ng/ml), cholera toxin (50 ng/ml) and hydrocortisone (500 ng/ml). The population doubling times of JIMT-1, L56Br-C1, MCF-7 and MCF-10A cells under these conditions were, approximately 24, 32, 34 and 15 h, respectively. The MCF-10A and JIMT-1 cell lines were sub-cultured twice a week, while the L56Br-C1 and MCF-7 cells were sub-cultured once a week with an additional change of growth medium once a week. For all experiments, the cells were seeded and allowed to attach and grow for 24 h, before addition of NSpd, Pd-NSpd or Pt-NSpd.

### Drug Stock Solutions

Stock solutions (2 mM) of NSpd were made in PBS, sterile-filtered and stored at 4°C. Pd-NSpd and Pt-NSpd were dissolved in 4% DMSO in PBS to give a stock solution of 2 mM, sterile-filtered and stored at –20°C. Further dilutions were made in complete cell culture medium to give the final concentrations. In all the experiments performed, aminoguanidine (1 mM) was added to the culture medium in order to inhibit the action of polyamine oxidase present in the serum.

### Dose-response Assay

The MTT assay was performed as previously described [35]. Briefly, cells were trypsinized, counted in a hemocytometer, pelleted and resuspended in cell culture medium. Aliquots of 180 µl cell suspension containing 3000 (MCF-10A), 5000 (JIMT-1) or 8000 (L56Br-C1 and MCF-7) cells were seeded in 96-well plates. At 24, 48 and 72 h of drug treatment, 20 µl of MTT solution (5 mg/ml in PBS) was added to each well and the 96 well plates were returned to the CO_2_ incubator for 1 h. The blue formazan product formed by reduction in live attached cells was dissolved by adding 100 µl of 100% DMSO per well. The plates were swirled gently at room temperature for 10 minutes to dissolve the precipitate. Absorbance was monitored at 540 nm using a Labsystems iEMS Reader MF (Labsystems Oy, Helsinki, Finland) and the software DeltaSoft II v.4.14 (Biometallics Inc., Princeton, NJ, USA). The results are presented as percentage of control.

### Cell Proliferation

Cells were seeded in Petri dishes (5 cm diameter) at a density of 0.3×10^6^ cells/Petri dish (MCF-10A cells) or 0.6×10^6^ cells/Petri dish (JIMT-1, L56Br-C1 and MCF-7 cells). The cells were harvested by trypsinization and the cell number was determined by counting in a hemocytometer.

### Effect of Long-time Treatment on Cell Proliferation

Cells (0.3×10^6^ MCF-10A cells, 0.7×10^6^ JIMT-1 cells, 0.7×10^6^ L56Br-C1 cells and 0.7×10^6^ MCF-7 cells) were seeded in replicates into 5 ml of medium in 25 cm^2^ cell culture flasks and the drugs were added to the final concentration of 25 µM. Seventy-two h later, the drug-containing medium was aspirated and fresh culture medium was added to the cells. After an additional 72 h, the cells were harvested by trypsinization and counted in a hemocytometer. The cells were reseeded at the same density as at the previous passage. The total recovery time between treatments was 96 hours. The cells received the same treatment during each treatment cycle and the experiment stretched over 5 weeks. The data are presented as the total amount of cells that theoretically would have accumulated if all cells had been reseeded with a known cell density after each treatment cycle. Thus, by using the cell number obtained at each passage of a culture seeded with 0.3 or 0.7×10^6^ cells (cell line dependent, please see above), we were able to calculate the number of cells that would have been obtained if all cells were reseeded at a lower density at each passage.

### Ornithine Decarboxylase Activity Assay

The cells were sonicated in ice-cold 0.1 M Tris-HCl (pH 7.5) containing 0.1 mM EDTA and 2.5 mM dithiothreitol and then ODC activity was determined in the sonicates by measuring the release of ^14^CO_2_ from carboxyl-labelled L-ornithine, in the presence of saturating levels of pyridoxal 5-phosphate (0.1 mM) and L-ornithine (0.2 mM), as previously described [52].

### ^3^H-spermidine Uptake Competition Assay

The effects of NSpd, Pd-NSpd or Pt-NSpd on uptake of ^3^H-Spd were studied in JIMT-1, L56Br-C1, MCF-7 and MCF-10A breast cells.

Cells (0.1×10^6^ cells per well) were seeded into 1.5 ml of medium in 12-well plates. Forty-eight h after seeding, the medium was removed and 1 ml new medium containing 2% FCS, 1 µM ^3^H-spermidine (0.5 Ci/mmol) and NSpd, Pd-NSpd or Pt-NSpd (0–10 mM) was added to the wells. The cells were incubated for 30 minutes at 37°C and then washed three times with PBS, followed by addition of 200 µl of 1 M NaOH. The plates were swirled gently at room temperature for 10 minutes and incubated at 37°C for 1 h, followed by addition of 200 µl of 1.5 M HCl. Afterward, the plates were again swirled gently at room temperature for 5 minutes, whereupon the radioactivity was measured in an aliquot using scintillation counting. The results are presented as percentage of control.

### Cell Cycle Phase Distribution and Cell Death Analysis by Flow Cytometry

Cells were seeded as for the proliferation assay and the drugs were added to the final concentration of 100 µM. Attached and floating cells were harvested, pelleted, fixed in 70% ice-cold ethanol and stored at –20°C until analysis. One day prior to analysis, cells were washed with PBS, pelleted and then incubated with PI-nuclear isolation medium (PBS containing 100 µg/ml PI, 0.60% Nonidet P-40 (NP-40) and 100 µg/ml RNase A) overnight at 4°C [53]. Immediately preceding the flow cytometry analysis, the cell suspension was suctioned three times through a syringe and filtered through a 50 µm nylon mesh. The cells were evaluated using an Ortho Cytoron Absolute flow cytometer (Ortho Diagnostic Systems, Raritan, NJ, USA) equipped with a 15 mW air-cooled argon-ion laser. For the computerized analysis of the cell cycle phase distribution and the sub-G_1_ region, the MultiCycle® software program (Phoenix Flow Systems, CA, USA) was used.

### Cell Cycle Kinetics and Analysis of BrdUrd and DNA Contents by Flow Cytometry

In order to evaluate effects on cell cycle progression, a DNA bromodeoxyuridine (BrdUrd) flow cytometric method was performed [54].

The cell lines MCF-10A and JIMT-1 (0.4×10^6^cells) and L56Br-C1 and MCF-7 (1.3×10^6^ cells) were seeded in 12 ml of medium in Petri dishes (9 cm in diameter) and grown in the presence of 25 µM NSpd, Pd-NSpd or Pt-NSpd for 72 h, whereupon BrdUrd was added to a final concentration of 5 µM. After 30 minutes of BrdUrd labeling, the medium was removed and the cells were rinsed twice with medium containing 0.5% FCS (37°C). The cultures were then further incubated with complete fresh medium (37°C) with and without 25 µM NSpd, Pd-NSpd or Pt-NSpd. The cells were harvested by trypsinization 0, 3, 6, 9 and 12 h post-labeling. The cells were pelleted, resuspended and fixed in ice-cold 70% ethanol and stored at −20°C until further analysis. The cells were prepared for analysis of DNA and BrdUrd contents and calculations were made as previously described [37], [38].

### Colony Formation in Soft Agar

Cells were incubated either in the absence or presence of 25 µM NSpd, Pd-NSpd or Pt-NSpd for 72 h before reseeding in soft agar at low density. After trypsinization and counting, the cells were resuspended in medium containing 0.3% agarose and 0.5 ml containing 500 cells was added to each well of 48-well plates coated with polyHEMA [55]. The cells were incubated at 37°C in a humidified incubator with 5% CO_2_ in air for 14 days and colonies were counted in an inverted phase contrast microscope.

### Intracellular Pd and Pt Accumulation

For the investigation of the intracellular accumulation of Pd-NSpd or Pt-NSpd, cells (1×10^6^ MCF-10A cells and 2×10^6^ JIMT-1 and L56Br-C1 cells) were seeded in triplicates into 12 ml of medium in Petri dishes (9 cm diameter) and the drugs were added to the final concentration of 25 µM. After 72 h of treatment, cells were washed three times with ice-cold PBS, harvested by trypsinization, counted, pelleted by centrifugation and stored at −80°C until analysis. Prior to analysis, the pellets were digested in 65% HNO_3_ for 2 h at 65°C, diluted to a 5% acid solution and centrifuged at 600 *g* for 14 min. The Pd and Pt accumulation was then analyzed by ICP-MS (Thermo X7, Thermo Elemental, Winsford, UK), as previously described [56]. The data of metal content was used to calculate the intracellular concentration of the compounds (Pd-NSpd or Pt-NSpd).

### Anthem’s in vitro Reporter-based Genotox Assay Using Genetically Engineered HCT116 Cells

The Genotox early-sensor stable cell line was derived from p53-competent human colon carcinoma, HCT116 cells. A single cell clone of this cell line was generated by serial dilution method and is referred to as HCT-p21-GADD-p53 herein, using lentiviral vectors carrying the Genotox early-sensor cassettes. The cassettes included p21 promoter operatively linked to Renilla luciferase reporter gene, GADD153 promoter operatively linked to firefly luciferase reporter gene and p53 response elements operatively linked to β-galactosidase reporter gene. The cell line was maintained in DMEM media (Sigma, USA) supplemented with 8% heat-inactivated FCS (Biowest, USA) and 100 U/ml penicillin G sodium and 100 µg/ml streptomycin sulphate at 37°C with 5% CO_2_.

Shortly, 1.3×10^4^ Genotox early-sensor HCT116 cells were seeded per well in a 96-well plate 16 h before test compound treatment. The cells were incubated in triplicates with five concentrations (1, 10, 25, 50 and 100 µM) of NSpd, Pd-NSpd or Pt-NSpd. Aminoguanidine hemisulfate was added to the FCS containing cell culture medium to a final concentration of 1 mM. Cells treated with methyl methanesulfonate (400, 200 and 100 µM) were included as assay positive control and vehicle-treated (1% DMSO) cells as negative control. At 72 h post-incubation, the cells were gently washed twice with PBS and lysed using lysis buffer (750 mM HEPES, 1.25% Triton X-100, 5 mg/mL porcine gelatin, 50% glycerol and 0.25% antifoam). The cell lysates were processed for renilla luciferase, firefly luciferase and β-galactosidase reporter assays. Briefly, 20 µl of the cell lysate was reacted with substrates (coelentrazine and D-luciferin from Nanolight technologies, USA, for Renilla luciferase, and firefly luciferase, respectively and ortho-nitrophenyl-β-galactoside from Sigma, India for β-galactosidase) and assay buffers (Luciferase assay buffer: 1 M Tricine, 0.5 M MgSO_4_, 0.5 M EDTA and 1 M DTT; β-galactosidase buffer: 60 mM Na_2_HPO4, 60 mM NaH_2_PO_4_, 10 mM KCl and 1 mM MgSO_4_) in white luminometer plates (for luciferase assays) or absorbance plate (for β-galactosidase activity assay). The assay plates were then read using a BioTek plate reader and the raw data were recorded. The results are expressed as average fold induction of reporter gene expression in comparison to the vehicle treated controls. The test compound is considered as genotoxic if the average fold reporter gene induction at any of the tested dose is over 1.5 fold (*i.e.* a 50% increase compared to vehicle treated controls).

### Single Cell Gel Electrophoresis Assay

The SCGE or Comet assay is a method used for detection of DNA damage in single cells. This method is used to evaluate DNA damage and repair, biomonitoring and genotoxicity [57]. The SCGE was performed with L56Br-C1, JIMT-1 and MCF-10A cells, as previously described [58], [59]. L56Br-C1 cells (0.7×10^6^ cells), JIMT-1 cells (0.7×10^6^ cells) and MCF-10A cells (0.3×10^6^ cells) were seeded into 5 ml of medium in Petri dishes (5 cm in diameter) and the drugs were added to the final concentration of 25 µM. Cells were harvested after 72 h of treatment and the cell number was determined by counting in a hemocytometer. For each sample, a total of 207 cells were analyzed for tail length and % DNA in tail using Comet Score™ Freeware (TriTek Corp. USA) and tail moment (TMOM: tail length multiplied by % DNA in tail) was calculated for individual cells. TMOM is used as a DNA damage indicator. The experiment was repeated three times.

### Statistical Analysis

For the statistical evaluation, a two-tailed Student’s *t*-test was used. For the statistical evaluation of the cell cycle kinetics and highest mean TMOM value, a 1-way ANOVA followed by the Newman-Keuls Multiple Comparison test was used. Differences were considered statistically significant at p<0.05.

## Supporting Information

Figure S1 **Phase contrast images of JIMT-1, L56Br-C1, MCF-7 and MCF-10A cells treated with NSpd, Pd-NSpd or Pt-NSpd.** Twenty-four h after seeding the cells, NSpd, Pd-NSpd or Pt-NSpd was added to give a final concentration of 25 µM. After 72 h of treatment, the cells were photographed with a digital camera attached to a phase contrast microscope. The L56Br-C1 cultures were rinsed with PBS to remove all dead cells floating, which were prominent in NSpd- and Pd-NSd-treated cultures where attached cells were found in small groups seen in the images.
(TIF)

Figure S2 **Representative histograms of the cell cycle phase distribution of JIMT-1, L56Br-C1, MCF-7 and MCF-10A cells treated with NSpd, Pd-NSpd or Pt-NSpd.** Twenty-four h after seeding the cells, NSpd, Pd-NSpd or Pt-NSpd was added to give a final concentration of 100 µM. After 72 h of treatment, the cells were harvested by trypsinization and the cells nuclei were stained with propidium iodide and analyzed by flow cytometry.
(TIF)

Figure S3 **The single cell gel electrophoresis (SCGE) assay was used to evaluate DNA damage in JIMT-1 cells.** Twenty-four h after seeding of JIMT-1 cells, NSpd, Pd-NSpd or Pt-NSpd was added to give a final concentration of 25 µM. After 72 h of treatment, cells were harvested for SCGE analysis. The ethidium bromide-stained nucleoids were photographed and then examined using the Comet Score™ Freeware. **A**. Images of comets obtained by the SCGE assay. DNA damage results in comets with head and tail, whereas undamaged DNA results in a round head. **B**. Percentage DNA in tail on the x-axis *versus* tail length on the y-axis for individual cells. **C**. Tail moment TMOM (%DNA in tail multiplied by tail length) for individual cells. Data were collected from three independent experiments, n = 207 cells. **D**. Table showing the mean TMOM value of the 10% highest TMOM values *i.e.* 20 highest values ± SD. *p<0.05 compared to control.
(TIF)

Figure S4 **The single cell gel electrophoresis (SCGE) assay was used to evaluate DNA damage in MCF-10A cells.** Twenty-four h after seeding of MCF-10A cells, NSpd, Pd-NSpd or Pt-NSpd was added to give a final concentration of 25 µM. After 72 h of treatment, cells were harvested for SCGE analysis. The ethidium bromide-stained nucleoids were photographed and then examined using the Comet Score™ Freeware. **A**. Images of comets obtained by the SCGE assay. DNA damage results in comets with head and tail, whereas undamaged DNA results in a round head. **B**. Percentage DNA in tail on the x-axis *versus* tail length on the y-axis for individual cells. **C**. Tail moment TMOM (%DNA in tail multiplied by tail length) for individual cells. Data were collected from three independent experiments, n = 207 cells. **D**. Table showing the mean TMOM value of the 10% highest TMOM values *i.e.* 20 highest values ± SD. ***p<0.001 compared to control.
(TIF)
