# Supplementary figures and images for: Norspermidine and Novel Pd(II) and Pt(II) Polynuclear Complexes of Norspermidine as Potential Antineoplastic Agents Against Breast Cancer
Source: PLoS One. 2013 Feb 13;8(2):e55651. doi: 10.1371/journal.pone.0055651 (PMC3572109; doi:10.1371/journal.pone.0055651)

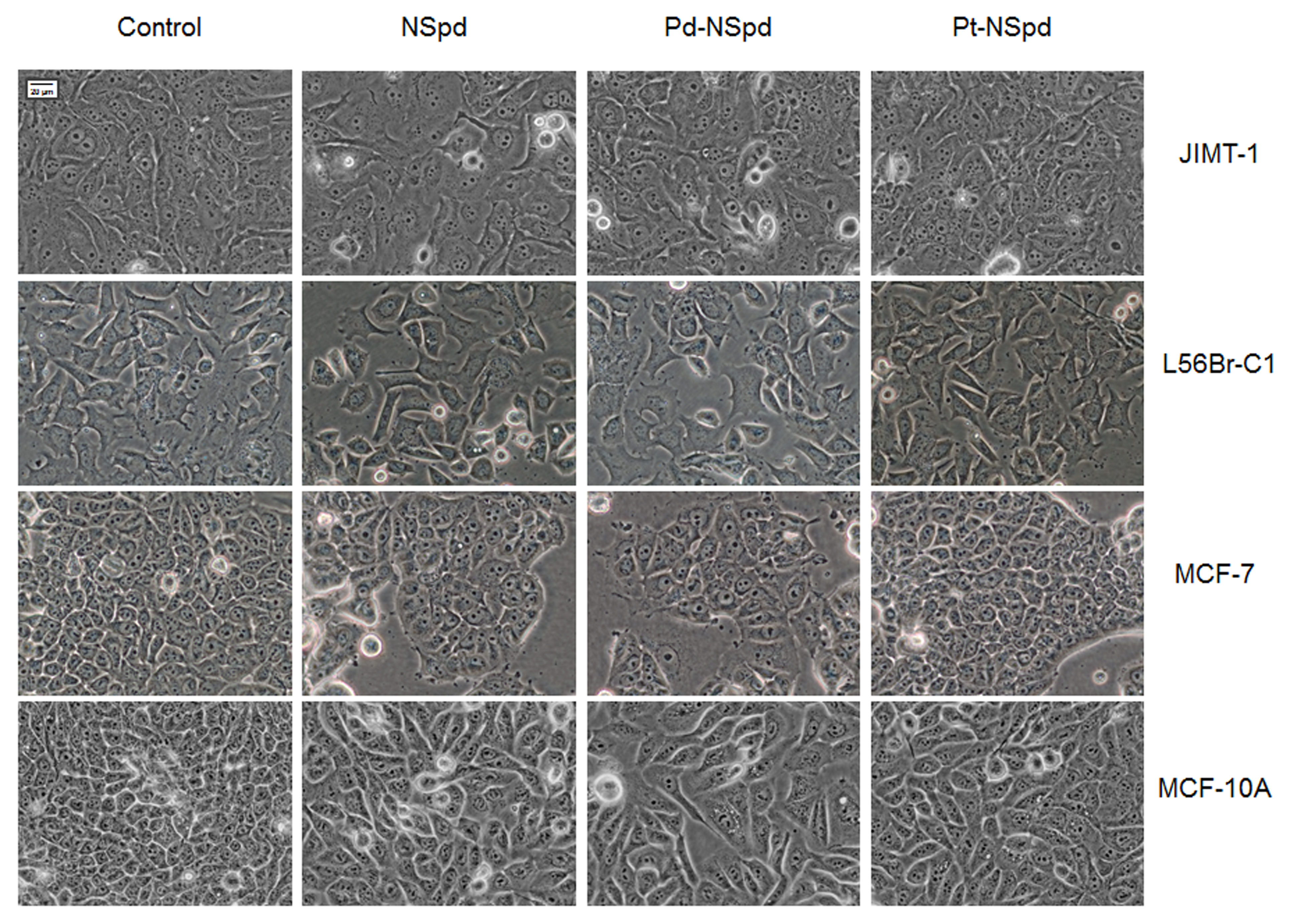

Supplement: Figure S1 — Phase contrast images of JIMT-1, L56Br-C1, MCF-7 and MCF-10A cells treated with NSpd, Pd-NSpd or Pt-NSpd. Twenty-four h after seeding the cells, NSpd, Pd-NSpd or Pt-NSpd was added to give a final concentration of 25 µM. After 72 h of treatment, the cells were photographed with a digital camera attached to a phase contrast microscope. The L56Br-C1 cultures were rinsed with PBS to remove all dead cells floating, which were prominent in NSpd- and Pd-NSd-treated cultures where attached cells were found in small groups seen in the images. (TIF) [file pone.0055651.s001.tif]

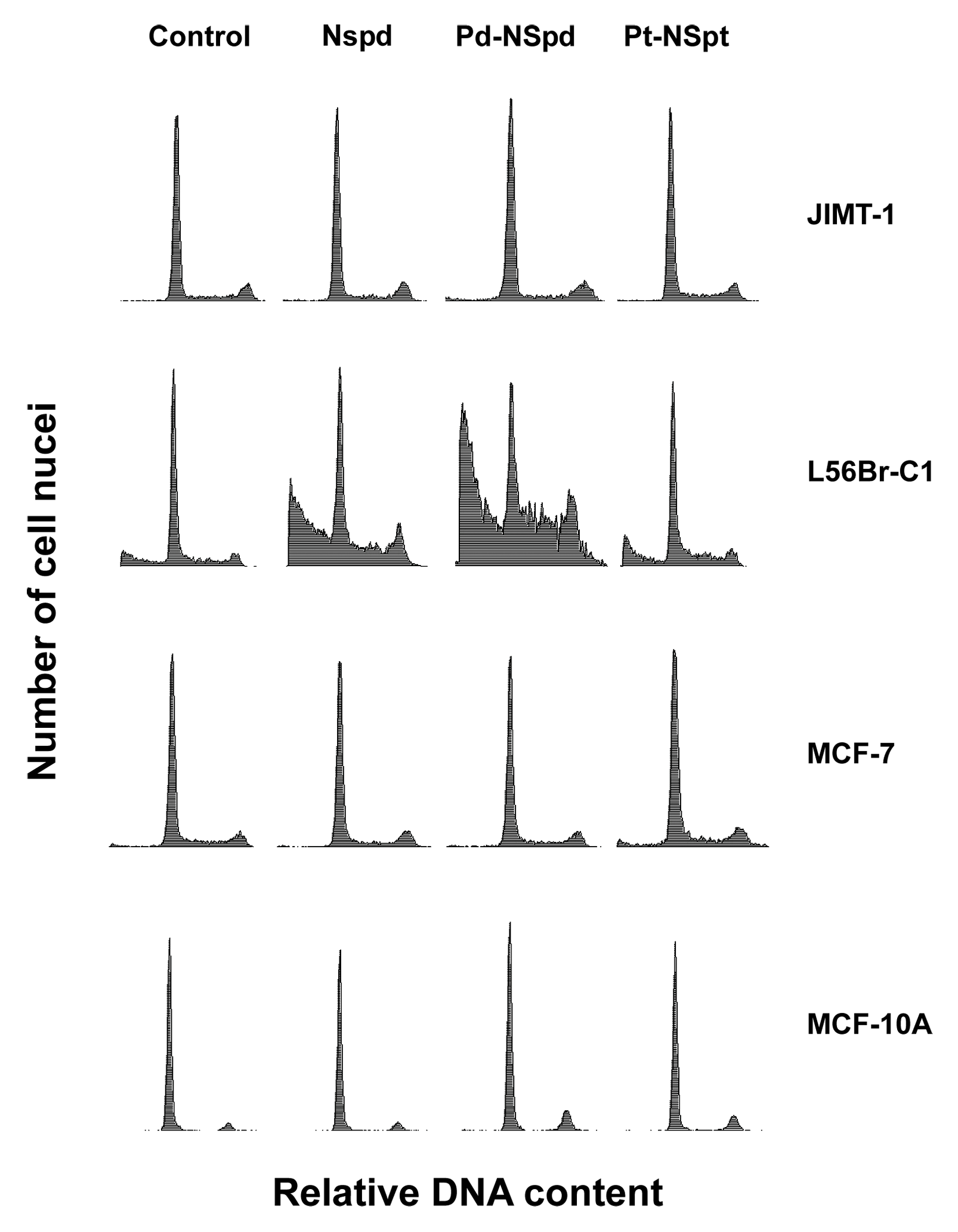

Supplement: Figure S2 — Representative histograms of the cell cycle phase distribution of JIMT-1, L56Br-C1, MCF-7 and MCF-10A cells treated with NSpd, Pd-NSpd or Pt-NSpd. Twenty-four h after seeding the cells, NSpd, Pd-NSpd or Pt-NSpd was added to give a final concentration of 100 µM. After 72 h of treatment, the cells were harvested by trypsinization and the cells nuclei were stained with propidium iodide and analyzed by flow cytometry. (TIF) [file pone.0055651.s002.tif]

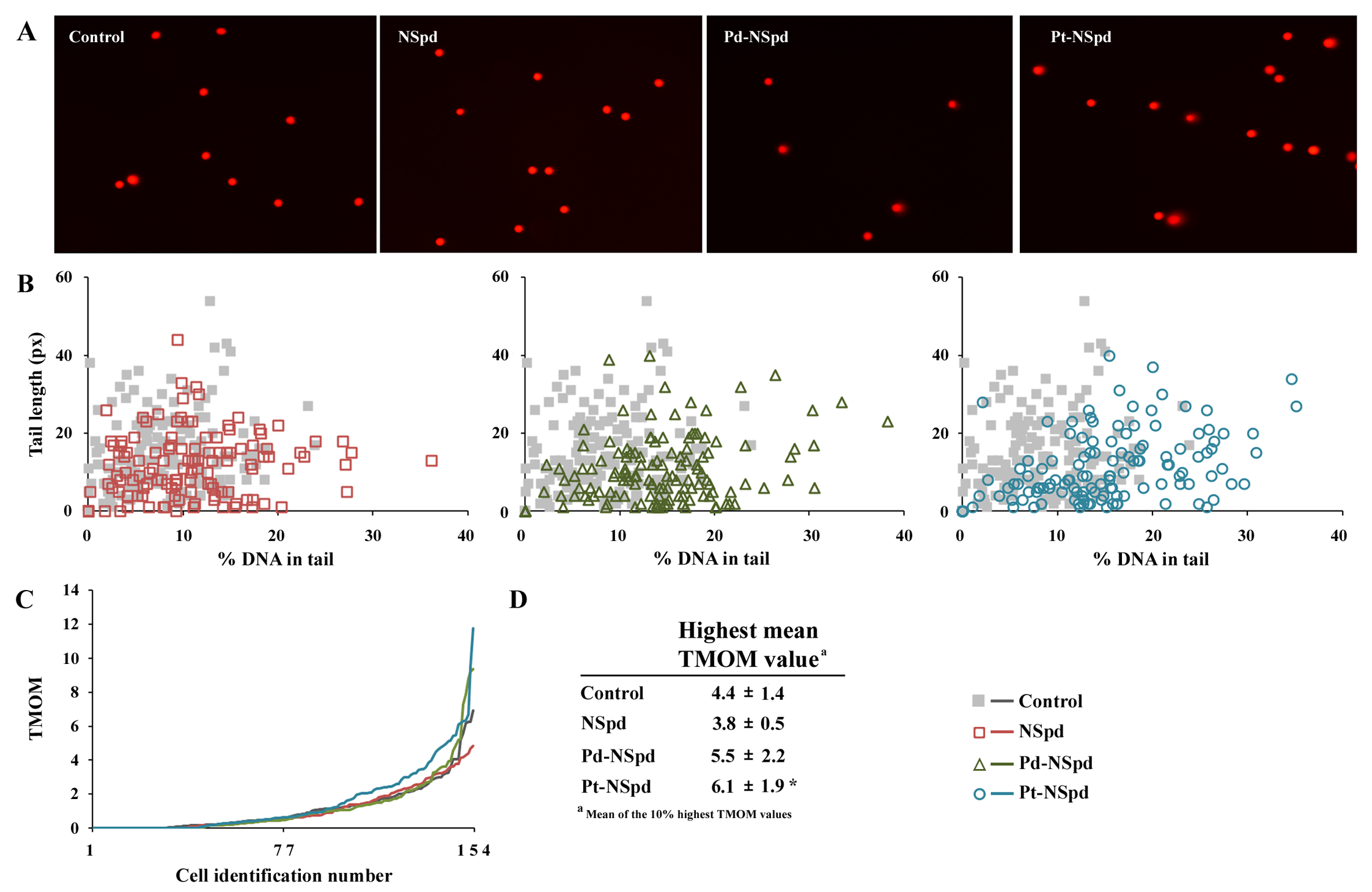

Supplement: Figure S3 — The single cell gel electrophoresis (SCGE) assay was used to evaluate DNA damage in JIMT-1 cells. Twenty-four h after seeding of JIMT-1 cells, NSpd, Pd-NSpd or Pt-NSpd was added to give a final concentration of 25 µM. After 72 h of treatment, cells were harvested for SCGE analysis. The ethidium bromide-stained nucleoids were photographed and then examined using the Comet Score™ Freeware. A. Images of comets obtained by the SCGE assay. DNA damage results in comets with head and tail, whereas undamaged DNA results in a round head. B. Percentage DNA in tail on the x-axis versus tail length on the y-axis for individual cells. C. Tail moment TMOM (%DNA in tail multiplied by tail length) for individual cells. Data were collected from three independent experiments, n = 207 cells. D. Table showing the mean TMOM value of the 10% highest TMOM values i.e. 20 highest values ± SD. *p<0.05 compared to control. (TIF) [file pone.0055651.s003.tif]

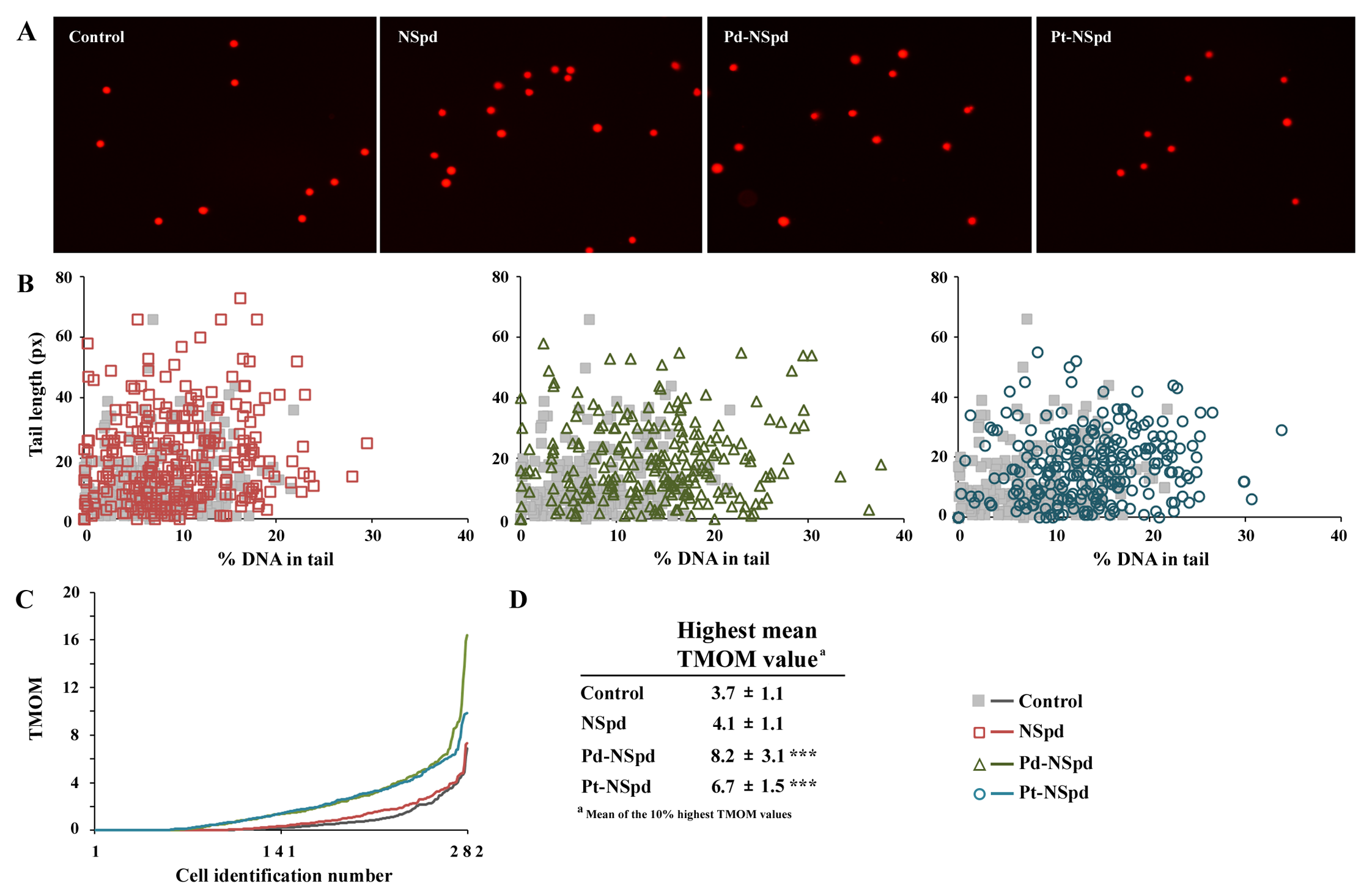

Supplement: Figure S4 — The single cell gel electrophoresis (SCGE) assay was used to evaluate DNA damage in MCF-10A cells. Twenty-four h after seeding of MCF-10A cells, NSpd, Pd-NSpd or Pt-NSpd was added to give a final concentration of 25 µM. After 72 h of treatment, cells were harvested for SCGE analysis. The ethidium bromide-stained nucleoids were photographed and then examined using the Comet Score™ Freeware. A. Images of comets obtained by the SCGE assay. DNA damage results in comets with head and tail, whereas undamaged DNA results in a round head. B. Percentage DNA in tail on the x-axis versus tail length on the y-axis for individual cells. C. Tail moment TMOM (%DNA in tail multiplied by tail length) for individual cells. Data were collected from three independent experiments, n = 207 cells. D. Table showing the mean TMOM value of the 10% highest TMOM values i.e. 20 highest values ± SD. ***p<0.001 compared to control. (TIF) [file pone.0055651.s004.tif]
